# Mindful regulation of positive emotions: a comparison with reappraisal and expressive suppression

**DOI:** 10.3389/fpsyg.2014.00243

**Published:** 2014-03-24

**Authors:** Fanny Lalot, Sylvain Delplanque, David Sander

**Affiliations:** ^1^Faculty of Psychology and Educational Sciences, University of GenevaGeneva, Switzerland; ^2^Distance Learning University of SwitzerlandBrig, Switzerland; ^3^Swiss Center for Affective Sciences, University of GenevaGeneva, Switzerland

**Keywords:** emotion regulation, mindfulness, reappraisal, expression suppression, positive emotion, facial expression, CERT

## Abstract

It is often acknowledged that mindfulness facilitates emotion regulation on a long-term scale. Only few empirical studies support the hypothesis that even a brief mindfulness induction among subjects without previous experience of meditation allows an effective reduction of both positive and negative emotions. To the best of our knowledge, this hypothesis has never been tested when comparing mindfulness to other regulation strategies known to be effective. The current study investigates the effects of mindfulness, reappraisal and expressive suppression during the regulation of positive emotions. Forty-five participants without previous meditation experience watched four positive video clips while applying a specific regulation strategy: mindful attention, reappraisal, expressive suppression or no strategy (control condition). Video clips were matched for intensity and positive emotions index. Each of them was evaluated on two dimensions, valence (negative/positive) and arousal (calming/exciting). Moreover, participants’ facial expressions were recorded during the presentation of the video clips. Results showed that (a) participants report less positive affect in reappraisal and mindful attention conditions compared to expression suppression and a control condition; and (b) the facialexpression – activation of AU12 (lip corner pull) and AU6 (cheek raiser) – varies with the regulation strategy applied. Results demonstrate the effectiveness of mindfulness in decreasing both the evaluative judgment of positive video clips and the related facial expression, among participants without previous mindfulness experience.

## INTRODUCTION

Emotion regulation can be defined as “attempts to influence which emotions we have, when we have them, and how these emotions are experienced or expressed” (Gross, 1998, pp. 224). One can rely on several regulation strategies to do so, although these strategies differ on some criteria. For example, the process model of emotion regulation (Gross, 1998, 2001; Gross and Thompson, 2007) highlights five families of strategies, depending on the point in the emotion-generative process at which the regulation takes place. So are distinguished: situation selection, situation modification, attention deployment, cognitive change, and response modulation.

Following Gross, most researches have focused on two strategies, namely expression suppression (a response modulation strategy) and reappraisal (a cognitive change strategy; e.g., Gross and John, 2003). Globally, the literature indicates that suppression is ineffective to reduce subjective feeling and physiological responses, and can even reinforce the latter (e.g., Gross and Levenson, 1993, 1997), supposedly because it takes place at a later stage in emotion generation (Gross and Thompson, 2007). By contrast, reappraisal has been found to effectively reduce subjective feeling as well as physiological responses (e.g., Ochsner and Gross, 2008). It has been proposed to be more effective than other strategies because it takes place early in emotion generation, before emotional reactions have fully unfolded (Richards and Gross, 2000).

In an alternative psychological account of emotion regulation, Koole (2009) lists more than 20 different strategies. The classification proposed by the author is based on two factors: an emotion-generating system (attention, knowledge, and bodily responses) and the function of the emotion regulation (satisfying hedonic needs [need-oriented], supporting specific goals pursuits [goal-oriented], and facilitating the global personality system [person-oriented]). According to this model, reappraisal is considered as a knowledge-based and goal-oriented strategy, while expression suppression is body-based and goal-oriented. Critically for the present study, Koole mentions mindfulness training as an attention-based and person-oriented strategy.

Whatever the theoretical framework adopted, it is worth noting that most researches focus on down-regulating negative emotions. However, the definition of emotion regulation includes possibilities of maintaining or even increasing any affect, including positive emotions (Gross, 1998). Indeed, there is evidence that regulation of positive affects is not only possible (e.g., Gross and Levenson, 1997; Ochsner and Gross, 2008; Reynaud et al., 2012) but also frequent (Nezlek and Kuppens, 2008). Studies on positive emotions almost always aim at up-regulating or maintaining them (e.g., Tugade and Fredrickson, 2007). This symmetry with negative emotions (typically down-regulated) indicates that the literature is dominated by a hedonic perspective. Tamir and Gross (2011) note that the classic approach of emotion regulation confines itself to investigating short-term consequences of emotions. By doing so, it “ignores the possibility that people may seek to regulate their emotions for reasons other than maximizing pleasure and minimizing pain” (Tamir and Gross, 2011, pp. 91). These authors defend a eudemonic approach of emotion regulation: they think that the human being aspires to achieve long-term well-being and happiness. To that end, one can punctually choose to increase one’s negative affect or decrease one’s positive affect, thus using emotions as means in goal pursuits and maximizing one’s functioning (Tamir, 2009). Within the psychopathology field, some consider the capacity for down-regulating positive emotions to be highly adaptive. Gruber (2011) suggests that positive emotion persistence is a disturbance that predicts bipolar disorder. Mania and addictions could also result from over-engagement with positive emotions (Hayes and Feldman, 2004).

A few studies have investigated the outcomes of the usual strategies on positive emotions. Reappraisal appears to be an effective strategy, allowing both an increase and decrease of positive affect (Kim and Hamann, 2007; Nezlek and Kuppens, 2008). Expression suppression has sometimes been found to effectively reduce self-reported positive affect as well as somatic activation (Gross and Levenson, 1997; Reynaud et al., 2012). However, some studies found this strategy to be ineffective (e.g., Korb et al., 2012) or obtained mixed results (e.g., Ortner and de Koning, 2013). Thus, there is some uncertainty about the effectiveness of suppressing positive affect.

Moreover, there appears to be gaps in the literature – particularly about the impact of other strategies than reappraisal and expression suppression. In the present study, we propose to investigate the effects of an alternative strategy on positive emotions: mindful attention. We hypothesize that mindfulness techniques can be used deliberately and efficiently to regulate positive emotions. We now briefly define the concept of mindfulness.

Mindfulness has been described as a process of paying attention in a specific way: on purpose, in the present moment, and non-judgmentally (Kabat-Zinn, 1994). Inspired from Buddhist meditation techniques, it was introduced in Western psychology with the mindfulness-based stress reduction program (MBSR; Kabat-Zinn, 1982). Practicing mindfulness typically consists of focusing on inner experiences (feelings, thoughts, and emotions) or on some aspects of the environment (pictures or sounds), with an attitude of non-judgmental acceptance: everything that enters awareness is “observed carefully, but is not evaluated as good or bad, true or false, healthy or sick, or important or trivial” (Baer, 2003, pp. 125).

Among numerous beneficial outcomes (see Brown et al., 2007, for a review), mindfulness has been shown to facilitate emotion regulation (e.g., Kabat-Zinn, 1994). Since the introduction of the MBSR, a considerable number of therapeutic interventions have integrated mindfulness training in their program, using it against stress, posttraumatic stress, anxiety, depression, borderline personality disorder, etc. (see Baer, 2003, for a review). The idea is that mindfulness, as a state of non-judgmental awareness, facilitates a healthy engagement with emotions, balancing between avoidance and over-engagement (Hayes and Feldman, 2004). Studies indicate a relationship between dispositional mindfulness, scores on the Difficulties in Emotion Regulation Scale (Erisman et al., 2009 in Chambers et al., 2009) and with the use of adaptive regulation strategies (Feldman et al., 2007 in Chambers et al., 2009). Mindfulness is also associated with greater emotion differentiation and less emotional difficulties (Hill and Updegraff, 2012). On this basis, Koole’s (2009) taxonomy integrates mindfulness as a person-oriented strategy, i.e., affecting one’s global personality system on a long-term scale.

Although the first studies investigated the effects of several weeks of mindfulness training (or of high dispositional scores), some recent studies have started investigating the effects of shorter inductions (a few minutes) on emotion regulation, among participants with no prior experience with meditation. Broderick (2005) induced a negative mood and then compared the effect of a 10-min episode of distraction, rumination and mindfulness among non-meditators. This short mindfulness exercise was shown to reduce negative mood, in comparison to the other strategies. Arch and Craske (2006) used a focused breathing induction and measured the duration participants tolerated looking at shocking pictures. Comparing mindfulness to unfocused attention and worrying, they found mindfulness led to lower negative affect. Papies et al. (2012) even found a diminished approach reaction to attractive food just by asking participants to consider food stimuli with mindful attention. Based on these pieces of evidence, we suggest that mindfulness can be used voluntarily and punctually to regulate a specific emotional episode.

Specifically, we hypothesize that one can work on the quality of the attention they allocate to a situation, therefore modifying one’s emotional response. This emotional response would be diminished, assuming that mindful attention hinders emotional over-engagement (Hayes and Feldman, 2004; Erisman and Roemer, 2010). It must be noted however that specific mechanisms through which this state of awareness influences emotional process are still unclear (e.g., Taylor et al., 2011).

According to Gross’ (1998, 2001; Gross and Thompson, 2007) model, mindful attention belongs to the “attention deployment” family, thus taking place earlier in the emotion generation process. Consistently, Koole’s (2009) terminology considers mindfulness an attention-based strategy. However, our hypothesis implies that it should not only be considered a person-oriented, long-term strategy but also a goal-oriented (i.e., effortful, voluntary, punctual) strategy.

In a recent review of mindful emotion regulation, Chambers et al. (2009) highlighted the differences between mindfulness and other strategies. Mindfulness would be antithetical to expression suppression, as the individual learns “to accept, rather than reflexively act on thoughts and emotions” (Chambers et al., 2009, pp. 566). It would also differ from reappraisal since in mindfulness, thoughts and emotions are considered simple mental states for which no action is required. On the contrary in the reappraisal perspective, thoughts and emotions “are treated as having some kind of inherent existence, and thus must be acted upon in some way (…). They can be changed to be more accurate or more psychologically beneficial representations of reality (hence reappraisals)” (Chambers et al., 2009, pp. 566). Despite these theoretical distinctions, empirical tests are scarce. To our knowledge, no study has compared mindfulness with reappraisal or suppression. Moreover, we lack evidence about the effects of mindful attention applied to positive affect.

Going back to Buddhism psychology, one can see that an excessive engagement in emotions is seen as problematic, whether these emotions are negative or positive. Even excitement and pleasure may lead to a false apprehension of reality (illusions) and to mental afflictions (Goleman, 2003). Thus, mindfulness should aim to decrease over-engagement in both positive and negative affect. As briefly described earlier, this Buddhist perspective is supported by Western authors (see Hayes and Feldman, 2004). Empirically, only a few studies have investigated the effect of a mindful attention on positive emotions. Taylor et al. (2011) had experienced vs. novice meditators watch positive, negative and neutral pictures with a mindful vs. “non-mindful” attention. Results showed that all pictures were evaluated as less intense in the mindful attention condition than in the control condition, among all participants. In their study already mentioned above, Arch and Craske (2006) also used positive stimuli. Participants reported less positive affect in the mindfulness condition, in comparison to the preoccupation condition. It must be noted that no differences were found between mindfulness and the control condition (unfocused attention). Nevertheless, this suggests that mindfulness does not increase positive affect (but see Erisman and Roemer, 2010). Considering that mindfulness consists of paying full attention to thoughts and emotions, one can speculate that the exercise actually increases the salience of feelings and in turn increases their subjective intensity. However, it is important to remember that this “full attention” must be characterized by a quality of acceptance and non-judgment. Thus, we suggest that mindful attention would decrease subjective positive feelings.

In the present study, we compare mindful attention with well-known strategies of reappraisal and expression suppression. In addition, a “no regulation” (control) condition is used as a baseline. We are measuring subjective positive affect induced by movies and related facial expressions. We expect mindful attention to be as effective as reappraisal in reducing subjective feeling as well as facial emotion expression.

## MATERIALS AND METHODS

### PARTICIPANTS AND DESIGN

Forty-five individuals (30 women, 15 men; 18–60 years of age, *M* = 27.3, SD = 10.2) with no prior experience of mindfulness meditation participated in the experiment. The study was a repeated-measures design, with Regulation strategy as a within-subject factor (no regulation vs. expression suppression vs. reappraisal vs. mindfulness). Gender showed no effect on any dependent variables, nor did age, despite the wide age range of the sample. The effects of the regulation strategies were unaffected by the inclusion of these variables as covariates in the analyses.

### PROCEDURE

Participants entered the experimental room individually. They were told that the aim of the study was to investigate how people react to movie clips, and how they could manage these emotional reactions. Participants gave their informed consent and were then presented with the instructions of the emotion regulation strategies: expression suppression, reappraisal and mindful attention, respectively. The experimenter made sure that participants understood the instructions and clarified if necessary.

Participants were then shown four video clips, each associated with different instructions (counterbalanced across participants), including the “no regulation” one. Films were presented on a computer running E-Prime 2.0 Pro (Psychology Software Tools, Inc.). Instructions appeared on the screen so that participants could read them over again before the video started. At the end of the clip, they were asked to rate the film on two dimensions: valence and arousal. This procedure was repeated four times. At the very end, participants answered a dispositional mindfulness scale. Participants’ facial expression was filmed during the whole experiment^1^.

### MATERIALS AND METHODS

#### Regulation strategies

The following instructions were given to the participants (translation from French).

Control condition: “We will now show you a video clip. We ask you to watch it carefully.”

Expression suppression: “We ask you to not show what you may experience while watching the video (sensations, thoughts, emotions). In other words, try to control all external manifestations of your state, so that someone watching you could not guess what you are experiencing.”

Reappraisal: “We ask you, while watching the film, to adopt the perspective of an observer of the scene, and not the perspective of one of the characters. Tell yourself you are not connected to these individuals. You can also imagine that the scene you are watching will then develop and lead to a somewhat different end. However, we ask you not to think about something else, unrelated to the movie. Stay focused on the scene that is presented to you.”

Mindful attention: “We ask you to pay attention to every reaction (sensations, thoughts, emotions) that may arise while watching, but at the same time, try to keep it distant. Observe that these reactions are nothing but momentary and temporary states of mind, which appear and disappear. Compare them to clouds in the sky: they move, lose their shape and disappear. Consider your sensations, thoughts and emotions the same way. Observe your reactions without trying to change, suppress or avoid them.”

#### Films

Four video clips were selected from Schaefer’s database (Schaefer et al., 2010) based on several criteria: an induction of similar positive affect (positive scores derived from the Differential Emotions Scale between 3.51 and 4.18, on a 5-point scale) and of no negative affect (negative scores derived from the DES between 1.11 and 1.46, on a 5-point scale). Thus, the films were not ambivalent. Moreover, they were relatively similar in terms of arousal (arousal score between 4.67 and 5.66, on a 7-point scale). The clips were excerpts from the following movies: *When Harry Met Sally*, *The Dinner Game*,* Life is Beautiful*, and *The Dead Poets Society*. The first two were chosen to induce amusement and the two others to induce tenderness. Clips lasted from 100 to 228 s and were all set in French (dubbed version).

In order to counterbalance the association between films and instructions of regulation, four versions of the procedure were computed on this basis using Latin square. The order of presentation of the films was randomized.

#### Subjective evaluation

Participants were asked to judge the valence and the arousal level of the film on visual analog scales (600 pixels large) by moving the cursor along a horizontal axis with the mouse, from “negative” (left of the scale = 0) to “neutral” (middle of the scale = 300) to “positive” (right of the scale = 600) for the valence, and from “calm” (left) to “exciting” (right) for the arousal level. This measure represented subjective emotional experience.

#### Facial expression

Participants were told they would be filmed while watching the movies, notably to make sure that they respected the suppression instructions. Specific individual facial movements (i.e., action units related to smiling) were analyzed using an automatic system for coding facial expressions [Computer Expression Recognition Toolbox 5.1 (CERT 5.1)]. CERT is a software tool for fully automatic facial expression recognition. It codes the intensity of facial actions from the facial action unit coding system (FACS; Ekman et al., 1980, 2002) in real-time. Validation studies showed a good validity of this tool (for more details, see Littlewort et al., 2011). Two action units were chosen: AU6 (cheek raiser) and AU12 (lip corner puller). AU6 involves orbicularis oculi activity and AU12 involves zygomaticus major activity. Both are typically involved in the expression of joy.

The software recorded 5.5 to 6.5 frames by second, the variability was due to the processor activity. Consequently, a re-sampling transformation was applied to adapt the size of all files to 1’000 frames (Matlab,^®^ Mathworks). The average of all CERT output values (i.e., the likelihood of an AU being present that is proportional to the strength of the AU performance) across all frames, as well as the maximum CERT output values (i.e., the maximal strength of AU performance) were calculated for the selected action units.

#### Dispositional mindfulness scale

Participants completed a French version (Trousselard et al., 2010) of the Freiburg Mindfulness Inventory (FMI; Walach et al., 2006). The FMI is composed of 14 items on a 7-point Likert scale. Scores were normally distributed, *M* = 4.7, SD = 0.8, min = 2.6 and max = 6.1. However, no relations could be found between the FMI score and any of the dependent variables, nor was the score significant when introduced in the analyses as a covariate.

#### Manipulation checks

Participants evaluated the clarity of the instructions for each regulation strategy, just before playing the video. After watching and evaluating each film, they also indicated to which extent they thought they managed to follow the instructions. Both were assessed on 7-point Likert scales.

Overall, regulation instructions were rated as clear (*M* = 6.4, SD = 0.9). They were globally estimated to be respected (*M* = 5.0, SD = 1.2) but some more than others, *F*(2,127) = 7.0, *p *<0.01. Specifically, participants indicated respecting mindful attention (*M* = 5.2, SD = 0.18) and expression suppression (*M* = 5.1, SD = 0.18) more than reappraisal (*M* = 4.6, SD = 0.18), *Post hoc* LSD *p*s <0.05.

#### Hypotheses and planned analyses

***Subjective evaluation of the clips*.** We expect an effect of the regulation strategy on both arousal and valence ratings, with specific differences between the strategies. Since the effectiveness of mindfulness is the main interest of the study and that it has been strongly suggested that reappraisal is effective when applied to positive emotions (Kim and Hamann, 2007; Nezlek and Kuppens, 2008), we predict lower arousal and less positive valence judgments for both the reappraisal and the mindfulness conditions compared to the control condition – which we are using as a baseline. Concerning expression suppression, evidence is less clear, but following some antecedent studies (Korb et al., 2012), we expect it to be ineffective in decreasing positive affect. In this condition, arousal and valence ratings would thus be equivalent to the control condition. The planned analysis will consist of a contrast opposing, on the one hand, reappraisal and mindfulness, and on the other, suppression and control condition.

***Facial expression*.** Concerning facial emotional expression, we also expect an effect of the regulation strategy. We consider both mean activation and maximum activation of the action units of interest; we think that variations in the intensity of the expression would be illustrated by a decrease of the general degree of smiling during the video, as well as by a decrease of the maximal smile a participant would show. Once again, the control condition is used as a baseline. We expect a certain degree of facial expressions in this condition, to the extent that the participants enjoy the video clips. If the suppression instruction is respected, participants would not express any joy (no smile). Thus, the facial expression would be the lowest in this condition (i.e. “pokerface”). Reappraisal would lead to lesser facial expressions than the control condition (see Goldin et al., 2008; Kim and Hamann, 2012). Indeed, according to Gross’ (1998, 2001; Gross and Thompson, 2007) model, reappraisal takes place early in emotion generation. Given that it modifies the emotional response, it seems legitimate that it would also impacts facial expression. For similar reasons, we predict lesser facial expressions in the mindfulness condition. Mindfulness belongs to the “attention deployment” family and thus takes place as early as reappraisal in the emotion generation process. Interestingly, we could not find any study investigating the intensity of facial expressions during mindful regulation of affect, positive or negative. So, our specific contrast hypothesis is: strongest activation in the control condition, medium and equivalent activations in the reappraisal and mindfulness conditions and lowest activation in the suppression condition.

## RESULTS

### SUBJECTIVE EVALUATION OF THE FILMS: TEST OF THE HYPOTHESIS

#### Baseline differences between the films

According to Schaefer et al.’s (2010) database, the four excerpts should be equally positive and arousing. To make sure of this, we first conducted a MANOVA to check for baseline differences between the films on the ratings of both valence and arousal within the control condition (no regulation). Concerning arousal, the four films were not perceived as differently arousing, *F *<1, n.s. Average arousal judgments in the control condition varied from 348.3, SD = 34.5 (*The Dead Poets Society*) to 414.0, SD = 34.5 (*When Harry Meets Sally*), on the 600-point axis. Concerning valence, the films were globally perceived as positive: average valence judgments in the control condition varied from 394.3, SD = 19.3 (*Life if Beautiful*) to 516.5, SD = 20.2 (*The Dinner Game*), on the 600-point axis. However, valence judgments differed significantly between the films, *F*(3,39) = 9.6, *p* <0.001. Thus, the films were integrated as an independent variable in further analyses considering valence ratings.

#### Arousal judgments

To test our hypothesis, a repeated-measures ANOVA with regulation strategy as a within-factor was conducted. The effect was not significant, *F*(3,37) <1, n.s. Thus, arousal was not considered in further analyses.

#### Valence judgments

**FIGURE 1:**
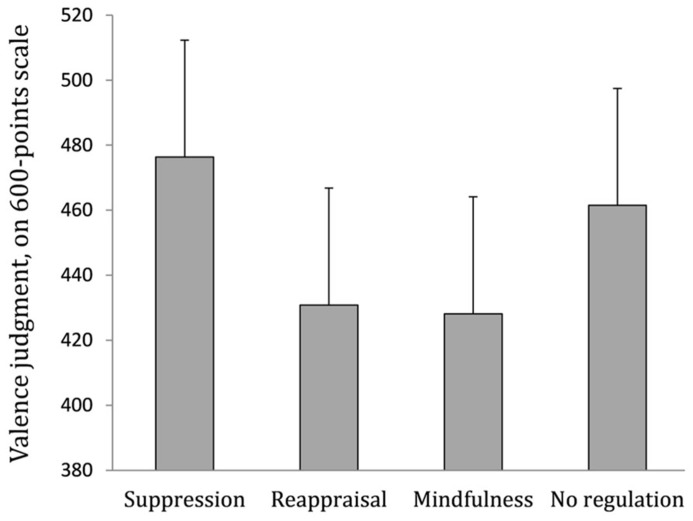
**Valence ratings depending on the regulation strategy condition.** A higher score represents a more positive valence judgment (300 = neutral judgment). Error bars indicate standard errors of mean.

Six observations, more than 2.5 SD under the mean, were excluded. A 4 (regulation strategy) × 4 (film) repeated-measures ANOVA revealed a main effect of the film, *F*(3,157) = 7.3, *p *<0.001, η^2^ = 0.12 and, more interestingly, a main effect of the regulation strategy, *F*(3,157) = 2.5, *p *= 0.05, η^2^ = 0.05. The following contrast was computed to test the main hypothesis: mindful attention = 1, reappraisal = 1, control condition = -1, suppression = -1; and revealed significance, *t* = -2.4, *p* = 0.02. Orthogonal contrasts found no difference between, on one hand, mindful attention, and reappraisal, *t* <1, n.s., and on the other, suppression and control condition, *t* <1, n.s. Thus, as predicted, valence judgments were less positive in conditions of reappraisal (*M* = 430.8, SD = 132.4) and mindful attention (*M* = 428.1, SD = 113.9) than in the suppression condition (*M* = 476.3, SD = 94.18) and control condition without regulation (*M* = 461.5, SD = 81.3). Means are illustrated in **Figure 1**. Finally, a film × regulation strategy interaction was also found, *F*(9,157) = 2.9, *p* = 0.004, η^2^ = 0.14.

### SUBJECTIVE EVALUATION OF THE FILMS: ADDITIONAL ANALYSES

As two different positive emotions (tenderness vs. amusement) were supposedly associated with the different films, we tested whether this factor would explain the interaction mentioned above. New contrast variables were computed for this purpose. The first contrast (C1) opposed the two amusement clips (*When Harry Met Sally* = -1 and *The Dinner Game* = -1) to the two tenderness ones (*Life is Beautiful* = 1 and *The Dead Poets Society* = 1). The second and third ones checked for differences between the two amusement clips (C2) and between the two tenderness clips (C3). An ANOVA was then conducted, considering these three contrast variables, the regulation strategy and the products between regulation strategy and each contrast variable. C1 did not reveal any significance, *t* = -1.12, *p* = 0.26, nor did its product with regulation strategy, *t* < 1, n.s. This indirectly showed that the type of emotion could explain neither the film main effect nor the valence rating of the film × regulation strategy interaction. Interestingly, C3, which opposed *When Harry Met Sally* to *The Dinner Game*, showed significance, *t* = -4.31, *p* < 0.001, as did the product of this contrast with regulation strategy, *t* = 4.40, *p* < 0.001.

Taking a closer look to the pattern of the film × regulation strategy interaction, it seemed that one film actually differed from the others, namely *When Harry Met Sally* (as suggested by the third contrast reported just before). The valence ratings of this excerpt went in opposite directions from the others (i.e., increasing in conditions of mindfulness and reappraisal and decreasing in suppression and control condition). Purely for information purposes, the initial regulation strategy × film ANOVA was conducted again, excluding *When Harry (…)*. The main effect of the film was still present, *F*(2,116) = 8.14, *p* < 0.001, as was the main effect of the regulation strategy, which showed even more reliability, *F*(3,116) = 5.38, *p* = 0.002. Gaps between the means were exacerbated: *M*_reappraisal_ = 404.6, SD_reappraisal_ = 138.7; *M*_mindfulness_ = 402.7, SD_mindfulness_ = 115.1; *M*_suppression_ = 480.8, SD_suppression_ = 93.1; *M*_control_ = 471.8, SD_control_ = 78.5. The initially significant film × regulation strategy interaction, was no longer significant: *F*(6,116) = 1.63, *p* = 0.15.

### FACIAL EXPRESSION OF EMOTION

**FIGURE 2:**
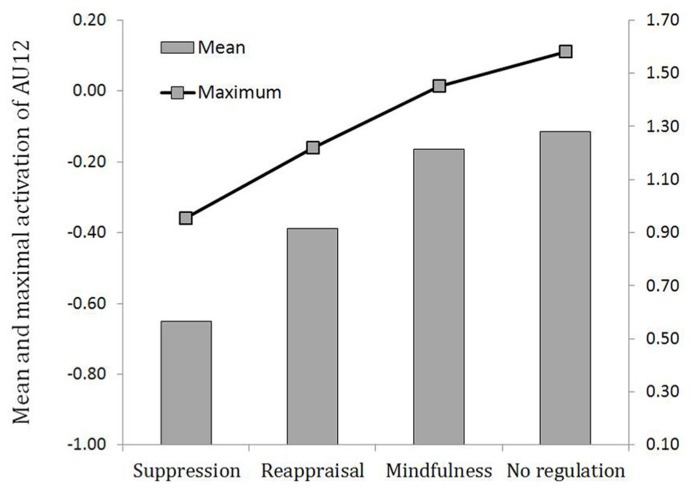
**Average and maximal activation of AU12 (lip corner puller) depending on the regulation strategy condition.** Left ordinate axis refers to average activation and right ordinate axis refers to maximum activation.

**FIGURE 3:**
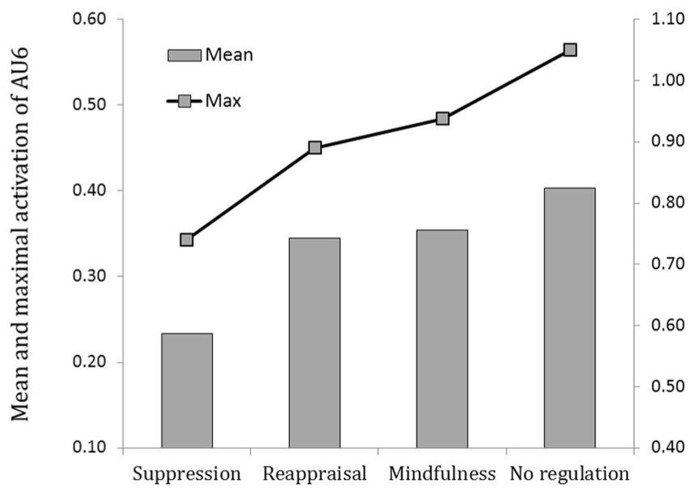
**Average and maximal activation of AU6 (cheek raiser) depending on the regulation strategy condition.** Left ordinate axis refers to average activation and right ordinate axis refers to maximum activation.

Three contrast variables were computed to test our hypothesis: C1 (suppression = -1; reappraisal = 0; mindfulness = 0; control condition = 1) and its orthogonal contrasts C2 (suppression = 0; reappraisal = -1; mindfulness = 1; control condition = 0) and C3 (suppression = -1; reappraisal = 1; mindfulness = 1; control condition = -1). A MANOVA tested the effect of the three contrasts on the different dependent variables, namely mean and maximum activation of both AU6 and AU12. C1 was found significant for all four dependent variables, *F*s(1,126) > 4.1, *p*s <0.045, while neither C2 or C3 reached significance, *F*s <1, n.s. This indicated that the mean intensity of the activation of both AU12 and AU6 was the lowest in the suppression condition, the highest in the control condition, and equivalently medium in the reappraisal and mindfulness conditions. The same conclusion applied to the maximal activation of AU12 and AU6. Means of the average and maximal activations are illustrated in **Figure 2** (AU12) and Figure **3** (AU6).

## DISCUSSION

The present study aimed to investigate the effects of mindful attention on positive affect, contrasted with reappraisal and expression suppression. The main hypothesis was that mindful attention would be effective, i.e., would lead to a decreased affect (operationalized through subjective evaluation in terms of valence and arousal) and a decreased facial expression (measured through the intensity of the activation of two facial action units associated with smiling: AU12 and AU6). Results showed that both mindful attention and reappraisal led to less positive valence ratings, compared to suppression and to the control condition without regulation. They also led to a lesser facial emotion expression (less smiling) than the control condition. The suppression condition, obviously, led to the least activation of the facial expression (“pokerface”).

For most emotion theorists, emotion is conceived as a synchronization of functionally defined components which produce an adaptive reaction to an event that is considered central to the individual well-being (see Sander, 2013, for a recent review). Those components are: the cognitive system responsible for the evaluation of the situation and the emotion determination, the autonomic system in charge of system regulation and physiological support, the motor system responsible for the expressive aspects, for communication of the reaction and behavioral intention, the motivational system is responsible for preparation and direction of action (approach/avoidance) and the monitor system in charge of subjective feeling. In the present study, we measured two of these components, namely subjective feeling and the motor system. Results indicated that these components do not necessarily covariate: under expression suppression condition, the motor system response was inhibited but the subjective feeling stayed activated. Future researches should adopt a multicomponential approach and investigate the activation of other components in parallel. Particularly, the cognitive component could inform about the recruited processes during regulation, and the physiological outcomes could indicate an impact on the mobilization of the organism’s resources.

Limitations to the present study include the fact that arousal and valence judgments were self-reported measures. It is possible that, after reading the different regulation instructions, the participants felt that we implicitly asked them to down-regulate their affect and thus report less positive affect, not because they actually felt less positive emotions but because they thought that was the correct answer. However, it should be remembered that under suppression instruction, which could have been understood as the most explicit down-regulation demand, participants reported positive valence judgments. For further studies, it would be beneficial to include more direct measures, like physiological measures (e.g., heart rate variability, skin conductance responses). Concerning the procedure, it must be noted that no training trials were included. Participants were directly confronted with the main task, without getting the opportunity to familiarize themselves with the regulation strategies. Though we only recruited participants without previous experience of mindfulness meditation, we did not control for other experiences, like yoga or psychotherapeutic treatments including mindfulness-based interventions. Given the numerous activities that involve a certain form of meditation, it seems impossible to control for everything. However, we did assess participants’ mindfulness level with the dispositional mindfulness scale and did not observe a significant influence on any of the strategies. Even if it appears unlikely that previous meditating experience have explained our pattern of results, further studies could compare non-meditators and meditators and investigate differences in the ability of mindfully regulating positive emotions. Concerning the material, we faced an important effect of the films on the evaluative variables, even though they should have been equivalent in terms of valence and arousal. The main issue concerned the *When Harry met Sally *excerpt, its pattern went in the opposite direction to the three other excerpts. On a methodological note, we could only recommend the use of multiple excerpts inducing the same emotion, to gain confidence in the results observed. Another methodological concern is the fact that the films we used were well-known to the general population. Participants report that they already know the clips in 65% of cases. We tested whether this factor could impact the evaluation of the movies. The analysis approached significance, *F*(1,160) = 3.5, *p* = 0.064, suggesting that films were rated as slightly more positive when already known (*M* = 462.5, SD = 102.3 vs. *M* = 429.0, SD = 121.0). This could be explained by something as simple as the mere exposure effect (Zajonc, 1968). For further studies, it would be useful to replicate these effects with unknown films, although tested and validated films are often excerpts from famous movies.

On a final note, even though the reported effects were significant, effect sizes were small. This could be due to several factors such as the sample being relatively small, thus the small effect size could reflect a lack of power. The differences of the baseline valence judgments between the films may also have played a role in reducing the part explained by the regulation strategies. An alternative explanation could be that the exercise was too short and thus did not allow a clear dissociation between mindful attention and the other strategies. Our results should therefore be compared to those emerging from studies investigating several-weeks-training.

There is one ambiguity that our study cannot directly answer, namely the question of whether participants drew a distinction between mindfulness and reappraisal. Indeed, both strategies showed similar effects (reduced judgment of positivity and reduced facial expression). However, we would like to point out that all regulation instructions were rated as clear and that, more relevantly, mindfulness instructions were assessed as more respected than reappraisal instructions (more difficult). Thus, it seems that participants did draw a distinction between both strategies. Our results suggest that mindfulness and reappraisal regulation strategies lead to similar patterns of subjective feeling and motor system activation during the emotional episode. However, it cannot be concluded from our study whether these strategies recruited the same cognitive, autonomic and motivational processes. Future studies adopting a multicomponential approach could also help to solve this issue.

Interestingly, previous work has suggested a link between mindfulness and reappraisal. In one study, Jermann et al. (2009) found a relationship between the dispositional mindfulness score and the use of positive reappraisal. For some authors, the relationship between mindfulness and reappraisal is circular: “positive reappraisal and mindfulness appear to serially and mutually enhance one another, creating the dynamics of an upward spiral” (Garland et al., 2011, pp. 59). Emotional benefits of mindfulness practice such as mood regulation (Jermann et al., 2009), stress reduction (Garland et al., 2011), or burnout reduction (Gerzina and Porfeli, 2012) should thus be mediated by increases in positive reappraisal coping. More recent (correlational) studies support this idea (e.g., Garland et al., 2013; Hayes-Skelton and Graham, 2013). However, another work considers mindfulness and reappraisal as two separated concepts (see Chambers et al., 2009) and defines mindfulness practice as “increased attention to present moment experience with a non-judgmental attitude and no attempt to cognitively reappraise emotionally salient (…) stimuli” (Chiesa et al., 2013, pp. 86). Recently, Chiesa et al. (2013) reconciled the two angles. Reviewing a dozen of neuroimaging studies investigating mindfulness and emotion regulation, they concluded that both approaches are relevant but apply to different individuals. Among novice meditators, mindfulness training recruits prefrontal cortex activation (associated with reappraisal) when regulating an emotion; but among expert meditators, mindfulness training is associated with reduced activation of limbic regions (i.e., amygdala and striatum) in response to emotional stimuli. Thus, there is a changeover from a top-down to a bottom-up mechanism (Chiesa et al., 2013). During its initiation to mindfulness techniques, the individual using mindfulness to regulate their emotions does it in a top-down manner: with no hold on the emotional stimulus, they can only act after the emotion has occurred – thus one understands the relationship between mindfulness and reappraisal. Secondly, when the individual masters mindfulness, they are prepared so that the process of emotional elicitation is modified (bottom-up angle). At this point, mindfulness and reappraisal are two distinct regulation strategies. Directly comparing expert and novice meditators, Taylor et al. (2011) obtained similar results. It should be noted that this distinction has already been proposed by Buddhist authors: “Once one has gained enough experience, one reaches the last rung: even before an emotion arises, one is prepared not to let it so much domination and power. This step is linked with blossoming, a state of complete transformation where emotions (…) arise with far less strength” (Matthieu Ricard cited by Goleman, 2003, pp. 166). Thus, even though mindfulness and reappraisal instructions lead to similar effects in our study, it does not mean they were confounded or disrespected by participants.

The main finding of this study is that individuals with no previous experience of meditation reported less positive affect and showed less joy expression when asked to adopt a mindful perspective. Although one of variables (namely, arousal) did not show any difference between the conditions, the significant results are noteworthy given that we have been able to directly and empirically compare mindfulness, reappraisal and expression suppression which, to our knowledge, has never been done. We could show that mindfulness allowed a reduction of positive affect – a phenomenon that very few studies have investigated. This is a step forward in understanding the relationship between mindfulness practice and emotion regulation.

## Conflict of Interest Statement

The authors declare that the research was conducted in the absence of any commercial or financial relationships that could be construed as a potential conflict of interest.

## Footnotes

1 The study complies with the Declaration of Helsinki guidelines on human experimentation. It respects the ethical code relative to research fixed by the ethical committee of the Faculty of Psychology and Educational Sciences of Geneva.
